# Direct Determination of a Giant Zero-Field Splitting of 5422 cm^–1^ in a Triplet Organobismuthinidene by Infrared Electron Paramagnetic Resonance

**DOI:** 10.1021/jacs.4c14795

**Published:** 2024-12-16

**Authors:** Tarek Al Said, Davide Spinnato, Karsten Holldack, Frank Neese, Josep Cornella, Alexander Schnegg

**Affiliations:** & Helmholtz-Zentrum Berlin für Materialien und Energie, Albert-Einstein-Strasse 15, 12489 Berlin, Germany; # Max-Planck-Institut für Kohlenforschung, Kaiser-Wilhelm-Platz 1, 45470 Mülheim an der Ruhr, Germany; § Max Planck Institute for Chemical Energy Conversion, Stiftstrasse 34-36, 45470 Mülheim an der Ruhr, Germany

## Abstract

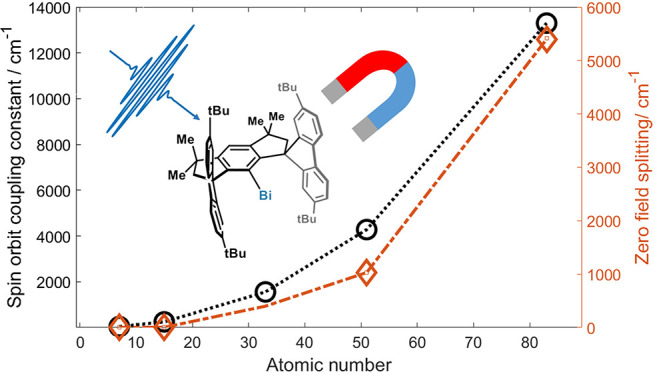

Stable monocoordinated organobismuthinidenes were only recently isolated and analyzed toward their chemical and electronic structure. Quantum chemical calculations on ^*t*^Bu-M^S^Fluind-Bi(I) (**2**) predicted an unusual electronic structure dominated by a triplet ground state and a spectacular zero-field splitting (ZFS) > 4500 cm^–1^. However, experimental evidence for these predictions remained elusive due to limitations in the available magnetic characterization techniques. Herein, we determine an axial ZFS of *D* = 5422 cm^–1^ for **2**, by direct detection of triplet electron paramagnetic resonance using magneto-optical infrared spectroscopy. To date, this represents the largest ZFS experimentally measured.

Bismuthinidenes are pnictinidene compounds (R–E, E = P, As, Sb, or Bi) with a monoanionic ligand (R), leaving the main group center in the formal +1 oxidation state. As a result, the compound bears two unpaired electrons in its outer p shell. In this context, organopnictinidenes and, in particular, organobismuthinidenes have recently received increasing attention due to their successful application as redox catalysts in challenging reactions.^1^

Depending on the substituent (R), organopnictinidenes can stabilize triplet (spin quantum number *S* = 1) electronic ground states.^3−10^ Pnictinidene triplet states exhibit large magnetic anisotropies (Figure 1) and are therefore of interest as building blocks in novel molecular magnets.^11,12^ Already the light nitrenes (R–N) were found to have the largest zero-field splitting (ZFS, quantified by the axial and rhombic ZFS parameters *D* and *E*, respectively) among all C-, N-, and O-centered polyradicals.^11^ Theoretical considerations revealed that the ZFS of nitrenes is dominated by spin–spin coupling between the two electron spins of the diradical.^5,13^ Interestingly, compounds containing several ferromagnetically coupled nitrenes can form quartet (*S* = 3/2), quintet (*S* = 2), and septet (*S* = 3) states with positive or negative ZFS.^11,14^ Electron paramagnetic resonance (EPR) studies on nitrenes^3,5,11^ and phosphinidenes^6,7,15^ allowed for a precise assignment of their magnetic anisotropies. The observed strong increase of the ZFS from nitrenes to the heavier phosphinidenes^7^ was explained by the increase of their SOC constants with *Z* (see Figure 1).

**Figure 1:**
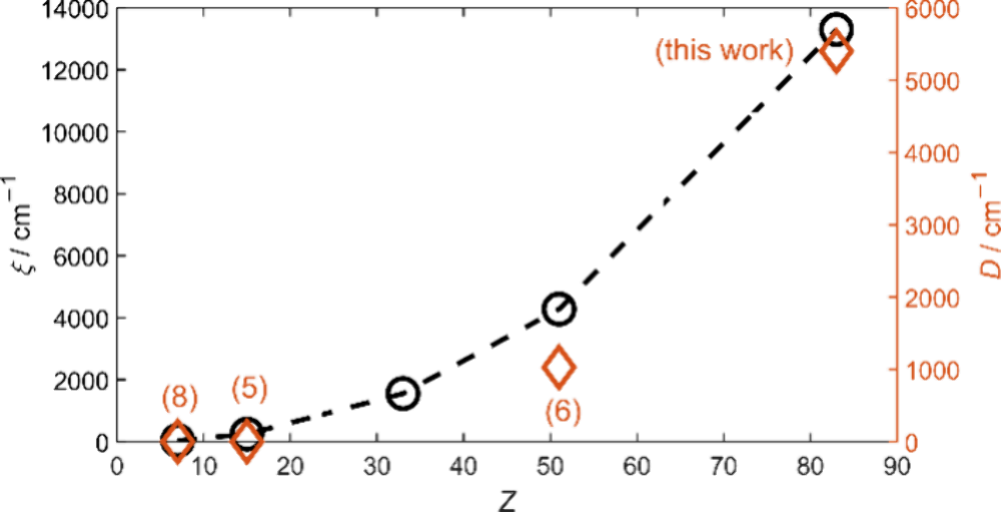
Calculated spin orbit coupling (SOC) constants^2^ ξ (black circles) and measured axial ZFS |*D|* (orange diamonds with references in parentheses) for selected triplet organopnictinidenes (see Table S1 in the Supporting Information (SI)) plotted vs atomic number *Z*.

The dramatic increase in magnetic anisotropy in triplet pnictinidenes with increasing *Z* is exciting and indicates enormous ZFS values for the heaviest triplet pnictinidenes, i.e., stibinidene (R–Sb) and bismuthinidene (R–Bi), exceeding even the very large magnetic anisotropies reported for some low-coordinate transition metal complexes^16^ and lanthanide single-molecule magnets.^17^ Large magnetic anisotropies are, next to the requirement of easy-axis anisotropy (i*.*e*.*, a negative axial ZFS), a necessary condition for magnetic hysteresis in single-molecule magnets. The blocking temperature below which single-molecule magnetism occurs depends on a characteristic energy barrier against magnetization reversal, which increases with the magnitude of the magnetic anisotropy.

Only a few reports are available for the heavy stibinidene (R–Sb)^8^ and bismuthinidene (R–Bi)^9,10,18^ on their synthesis and magnetic properties of their triplet states. For M^s^Fluind*-Sb, the only isolated organostibinidene, highly correlated wave function-based *ab initio* calculations predicted a triplet ground state with *D* = 940 cm^–1^.^8^ A combination of paramagnetic NMR (Evans method) and temperature-dependent susceptibility measurements (χ*T* vs *T*) with a superconducting interference device (SQUID) confirmed the triplet ground state and assigned an axial ZFS of *D* = 1030 cm^–1^.^8^ For the bismuthinidene ^*t*^Bu-M^s^Fluind-Bi(I) (**2**) (see Scheme 1), *ab initio* calculations also determined a ground state dominated by a triplet state with an even larger ZFS of *D* > 4500 cm^–1^.^10^ Within the present work, wave function based *ab initio* calculations using the complete active space self-consistent field (CASSCF) followed by second-order *N*-electron valence perturbation theory (NEVPT2) calculations in conjunction with the X2C relativistic Hamiltonian as implemented in the ORCA package^19^ (version 6.0) have been performed on **2**. These calculations confirmed the *S* = 1 ground state with nearly axial ZFS (*D* = 4523 cm^–1^ and *E*/*D* = 0.05) and yielded a *g*-matrix (***g***) with main values ***g*** = [1.77 1.84 1.98]. Further information on the calculations can be found in the SI. In the case of **2**, previous χ*T* vs *T* measurements showed hardly any susceptibility change upon increasing the temperature.^9,10^ These findings are in line with the assumption of a gigantic positive axial ZFS that leads to an energetically isolated *M*_*S*_ = 0 ground state. However, clear experimental evidence for this assumption has not been provided up to now since the magnetic characterization of triplet states with ZFS significantly above 1000 cm^–1^ poses severe technical difficulties. Fixed frequency EPR—the method of choice for a precise determination of ZFS in the GHz range—fails when the energy gap between the *M*_*S*_ levels induced by ZFS exceeds the excitation energy of the microwave quanta applied in the spectrometer (10 GHz/0.3 cm^–1^ for a standard X-band EPR spectrometer). This can be partly circumvented by moving to higher frequencies.^20^ However, the current limit for high-frequency EPR spectrometers is around 1 THz (30 cm^–1^), while for the predicted triplet ZFS of several thousand wave numbers EPR excitation energies in the same range would be required. SQUID magnetometry allows for robust assignment of integer spin states with ZFS up to several hundred cm^–1^. However, for ZFS *in the range of thousands of cm*^*–1*^ it is practically impossible to achieve a significant change of the *M*_*S*_ level population by the temperatures available in a SQUID magnetometer. Collectively, these challenges render the assignment of magnetic parameters increasingly difficult. At this point, we turned our attention to magneto-optical infrared (IR) spectroscopy. This technique probes electron-spin transition energies from the GHz to the mid-IR (2 cm^–1^ to 6000 cm^–1^), by far exceeding the excitation energies of conventional EPR spectrometers.

**Scheme 1:**
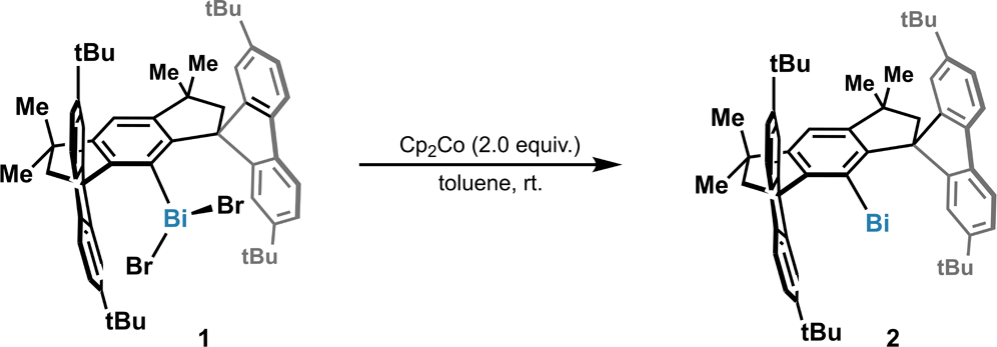
Structures of ^t^Bu-M^S^Fluind-BiBr_2_ (**1**) and ^t^Bu-M^S^Fluind-Bi(I) (**2**)

To identify resonant transitions within the triplet-*M*_*S*_ levels, we measured the broadband IR absorption of a sample placed in the variable-temperature inset of a high-field magnet. Electron-spin resonance peaks were discriminated from IR-active electric dipole transitions by comparing IR spectra recorded at different magnetic fields. The experiments were carried out on the setup shown in Figure S1 of the SI, which consists of a high-resolution Fourier transform infrared spectrometer (FTIR) connected to a superconducting high-field magnet by an evacuated quasi-optical transmission line.^21^

Figure 2 depicts the IR transmission spectra obtained on pressed powder pellets of paramagnetic **2** and its diamagnetic precursor compound **1** (cf. Scheme 1) at 10 K in the frequency range from 600 to 6000 cm^–1^. The spectrum shows the characteristic IR peaks reported for **1** and **2**.^10^ However, in the case of **2**, an additional absorption peak is observed at 5422 cm^–1^, which is absent in diamagnetic precursor complex **1**. To further characterize this peak, IR spectra of **2** were recorded at external magnetic fields of 5 and 10 T. Upon application of a magnetic field, a clear broadening of the peak at 5422 cm^–1^ was observed (Figure 3). Line broadening of the peak is already apparent at 5 T, but it is less pronounced (see Figures S4 and S5). The increased line broadening with increasing external magnetic field is attributed to the electron-spin Zeeman interaction, which increases linearly with an external magnetic field (see Figure S6 for a magnetic-field-dependent spinenergy level diagram). To analyze the magnetic field dependence, IR spectra taken at different field values are divided by each other. In Figure 3b, the corresponding 0 T/10 T IR magnetic-field division spectrum (IR-MDS) of **2** is shown. The noise in the spectra increases with increasing wavenumber since the transmitted radiation through the spectrometer decreases in this frequency range (Figure S2). As can be seen in Figure S5a, no further field-dependent IR resonance is observed in the spectral range from 3900 to 5600 cm^–1^. The resonance at 5422 cm^–1^ is therefore assigned to the EPR transition between the triplet *M*_*S*_ = 0 and *M*_*S*_ = ±1 levels (cf. Figure S6). Apart from the magnetic field dependent line broadening, no splitting of the IR peak is observed. A splitting of the EPR peak would be expected for a triplet state with rhombicity in its ZFS. Since this was not observed, the rhombicity of the ZFS is therefore assumed to be smaller than the fwhm line width of the IR resonance (2*E* < 40 cm^–1^ and *E*/*D* < 0.01). To test these assumptions IR-MDS were simulated with the frequency-domain simulation function of the Matlab toolbox EasySpin^22^ with the following triplet spin Hamiltonian (SH):
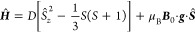
1

**Figure 2:**
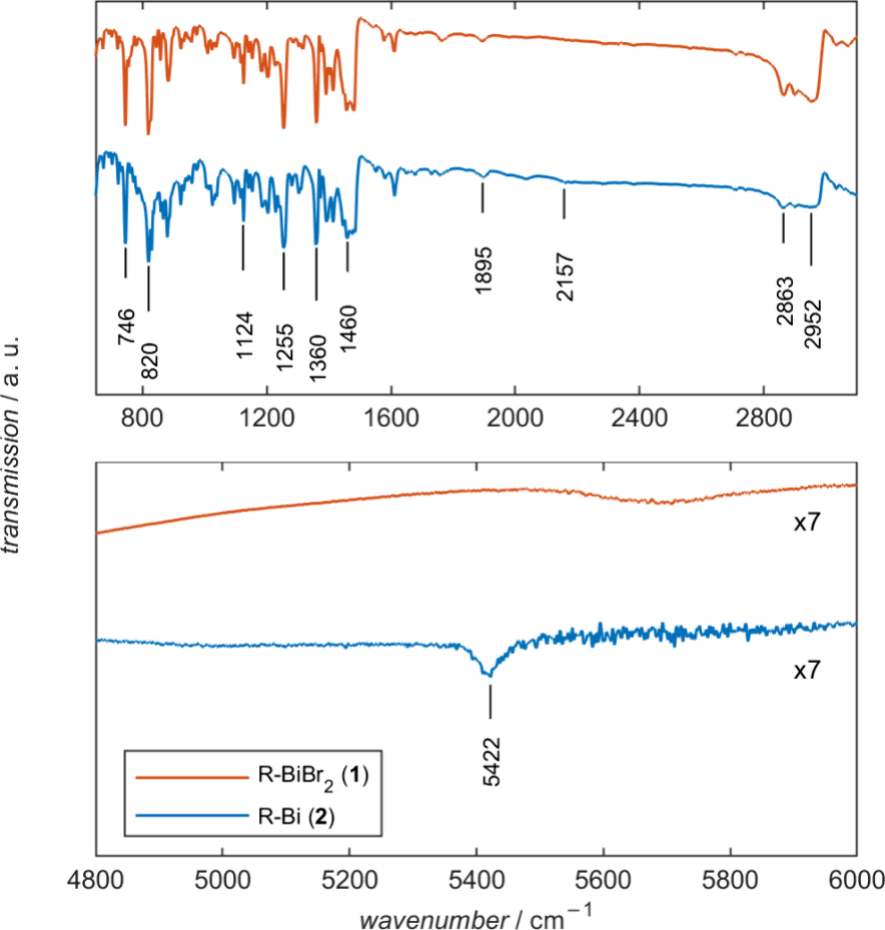
FTIR transmission spectra measured on powder samples of **1** (orange trace) and **2** (blue trace) at 10 K with a resolution of 2 cm^–1^. Spectra of **1** and **2** are vertically stacked for better visibility. The full FTIR spectrum is depicted in Figure S2.

**Figure 3:**
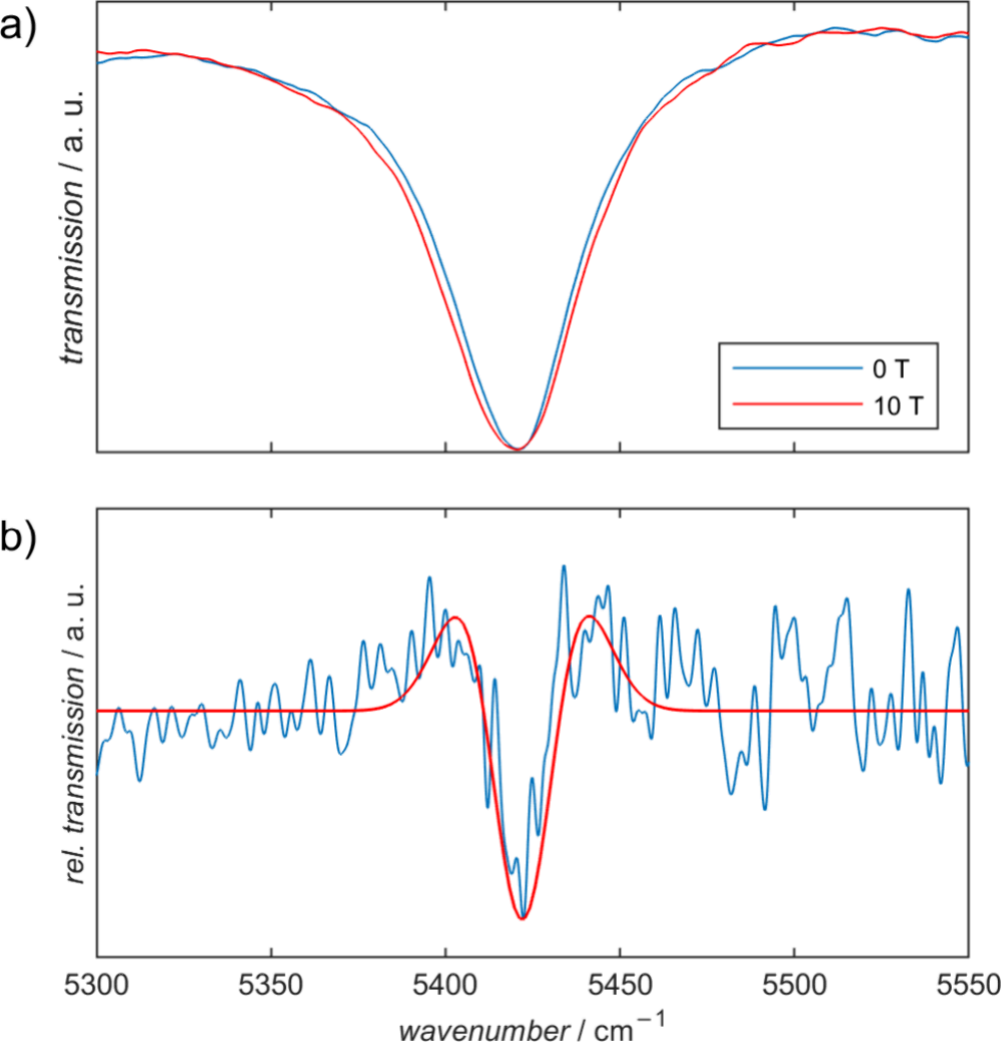
(a) Enlarged view of the IR transmission peak at 5422 cm^–1^ at 0 T (blue) and 10 T (red), b) experimental (blue) and simulated (red) IR-MDS. The blue trace was obtained by division of the spectra in the upper panel (0 T/10 T). Figure S5a shows the same IR-MDS plotted on a wider spectral range.

Here, the first term contains the second-order ZFS and the second term the electron-spin Zeeman interaction. For a triplet state with vanishing *E*, the transition energy between the *M*_*S*_ = 0 ground level and the degenerate *M*_*S*_ = ±1 levels corresponds to *D*. In the simulations, *D =* 5422 cm^–1^ was fixed to the center position of the IR peak and *E* was set to 0. Due to the limited resolution in the experimental IR-MDS an isotropic *g* = 2 was assumed (see Figure S7 for a comparison with simulations obtained with the ***g***-matrix from the *ab initio* calculations). With this model, IR-MDS for 0 T/10 T (Figure 3) as well as 5 T/10 T and 0 T/5 T (Figure S5) have been calculated. Very good correspondence between experiment and simulation was achieved, providing further strong evidence that **2** is largely dominated by a triplet ground state with a very large positive axial ZFS of *D* = 5422 cm^–1^.

The ground state electronic structure and the ZFS of **2** have been predicted by quantum chemical calculations.^9,10^ Indeed, the experimental evidence we provide here confirms the prediction of a triplet ground state with a ZFS of several thousand wave numbers; yet, the measured value was found to be ∼20% larger than the theoretical prediction. The magneto-optical IR experiments determine the ZFS directly from the position of the IR peak, with an uncertainty smaller than 1%. Hence, we assign the discrepancy between experimental and calculated *D*-values to the remaining inaccuracies in the theoretical model. This is not surprising, despite the impressive predictive power of the quantum chemical calculations and their ability to correctly predict the triplet character of **2** and the order of magnitude of its ZFS.

In conclusion, herein we employ IR detection of EPR resonances to provide direct experimental evidence of a positive large axial ZFS in **2**, with a *D* = 5422 cm^–1^ and *E*/*D* < 0.01. To the best of our knowledge, this is the largest ZFS ever measured experimentally for a given compound and places **2** as a landmark example in this field. With the described experimental setup, we demonstrate that ZFS up to several thousand wave numbers can be accurately determined by IR spectroscopy in combination with high magnetic fields. The possibility of predicting and measuring extremely large magnetic anisotropies significantly expands the opportunities of characterizing the emerging class of heavy pnictinidenes via magneto-structural correlations. Our results thereby open the door to the analysis of compounds with important implications in material science and molecular magnetism.

## Supporting Information Available

Details on the experimental setup, the spectral simulations, and the quantum chemical calculations as well as additional field-dependent IR measurements (PDF)

## ABBREVIATIONS

ZFS: (zero-field splitting)
EPR: (electron paramagnetic resonance)
SQUID: (superconducting interference device)
IR: (infrared)
IR-MDS: (infrared-magnetic division spectrum)
CASSCF: (Complete active space self-consistent field)
NEVPT2: (second-order *N*-electron valence perturbation theory)
