# Supplementary material for: Direct Determination of a Giant Zero-Field Splitting of 5422 cm–1 in a Triplet Organobismuthinidene by Infrared Electron Paramagnetic Resonance
Source: J Am Chem Soc. 2024 Dec 16;147(1):84–7. doi: 10.1021/jacs.4c14795 (PMC11726562; doi:10.1021/jacs.4c14795)
Supplement: Supplementary file 1 — ja4c14795_si_001.pdf [file ja4c14795_si_001.pdf]

Direct Determination of a Giant Zero-Field Splitting of 5422 cm^{-1} in a Triplet Organobismuthinidene by Infrared Electron Paramagnetic Resonance

Tarek Al Said,[&] Davide Spinnato,[‖] Karsten Holldack,[&] Frank Neese,[‖*] Josep Cornella, [‖*] Alexander Schnegg,[§*]

[&]Helmholtz-Zentrum Berlin für Materialien und Energie, Albert-Einstein-Strasse 15, 12489 Berlin, Germany
[‖]Max-Planck-Institut für Kohlenforschung, Kaiser-Wilhelm-Platz 1, 45470 Mülheim an der Ruhr, Germany
[§]Max Planck Institute for Chemical Energy Conversion, Stiftstrasse 34-36, 45470 Mülheim an der Ruhr, Germany

Supplementary Information

1) Sample Preparation

Samples were prepared by mixing compound **1** and **2** with potassium bromide and pressed to pellets of 10 mm diameter at 1000 psi under nitrogen atmosphere. Compounds **1** and **2** were synthesized according to the literature procedure (Ref. 1).

2) ZFS of Organopnictinidenes

Table S1 ZFS in triplet pnictinidenes with increasing atomic number *Z*.

Z	**SOC constant** [2]<br>ξ **/ cm**$^{-1}$	**Pnictinidene**<br>**/formula**	**ZFS / cm**$^{-1}$	**Ref.**
7	41. 9	Nitrene<br>1,3-diazido-2,4,6-tribromo-5-nitrenobenzene	D = 1.369 cm^{-1}<br>E/D = 0.068	3
15	247	Phosphinidene<br>triplet mesitylphosphinidene	D = 4.116 cm^{-1}<br>E/D = 0.001	4
33	1555	Arsinidene		
51	4280	Stibinidene<br>$\mathrm{M^{S}}$Fluind*-Sb(I)	D = 1030 cm^{-1}<br>E/D = 0.02	5
83	13300	Bismuthinidene<br>^{t}Bu-$\mathrm{M^{S}}$Fluind-Bi(I)	D = 4500 cm^{-1}<br>Calculated	1
			D = 4300 cm^{-1}<br>Calculated	6
			D = 4523 cm^{-1}<br>E/D = 0.05<br>Calculated<br><br>D = 5422 cm^{-1}<br>E/D < 0.01<br>Measured	Herein

3) Magneto-optical IR spectroscopy

Magneto-optical IR spectroscopy was performed at the THz-EPR beam-line at the BESSY II electron storage ring.[7] Figure S1 depicts a scheme of the set-up. For IR measurements on **1** and **2** a high-resolution Fourier-transform infrared (FTIR) spectrometer (Bruker IFS 125HR), was equipped with a glowbar IR source, a KBr beam splitter and a liquid-N_2-cooled mercury cadmium telluride (MCT) mid-infrared (MIR) detector (FTIR 16/MSL 12, Infrared Associates Inc.). A set of four IR transparent wedged diamond windows separate the high-vacuum in the FTIR spectrometer and the evacuated beam-line from the magnet vacuum and the latter from the variable temperature insert (VTI) of the superconducting high-field magnet (Cryogenics J4777) in which the sample is immersed. Magneto-optical IR transmission measurements were obtained with the spectrometer aligned in Voigt geometry (see Figure S1), with the magnetic-field component B_1 of the IR-radiation oriented perpendicular to the static magnetic field B_0 and with 2.0 cm^{-1} instrumental resolution. IR transmission spectra in Figure 2 of the main text were derived by dividing raw spectra of the samples by a raw spectrum of a plain KBr pellet recorded at identical

conditions. IR spectra in Figure 3 b of the main text and Figure S5 are shown as magnetic field division spectra (MDS), where raw spectra obtained at an external magnetic field B_0, were divided by spectra taken under identical conditions but different field (0 T, 5 T, 10 T).

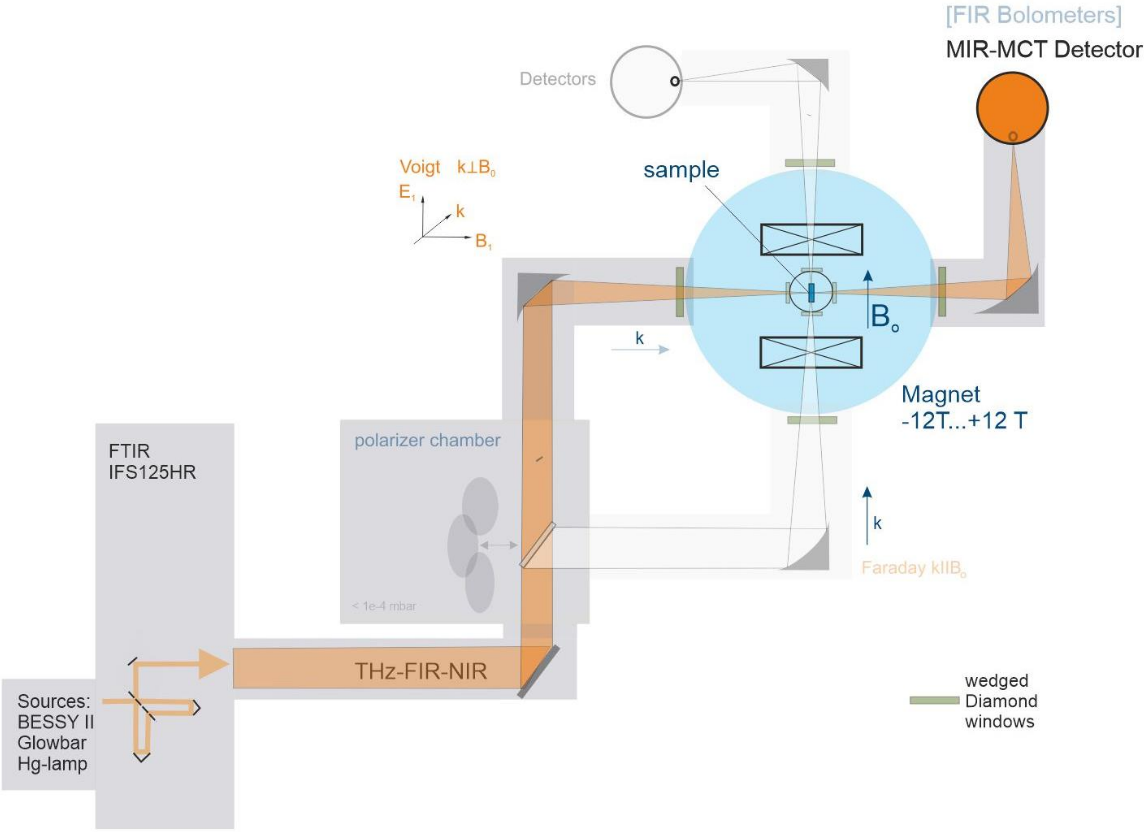

Figure S1. Magneto-optical IR set-up, where a high-resolution FTIR spectrometer is connected to a superconducting high-field magnet (light blue) and a LN_2 cooled MCT detector by a fully evacuated transmission line equipped with focusing optical mirrors. A blue rectangle indicates the sample position in the magnet. The path of the IR light is shown in orange. For the magneto-optical IR experiments on **1** and **2** unpolarized IR radiation was transmitted through the sample magnet in Voigt geometry (propagation direction (***k***) of the IR radiation oriented perpendicular to the direction of the external magnetic field ($\boldsymbol{B}_0$).

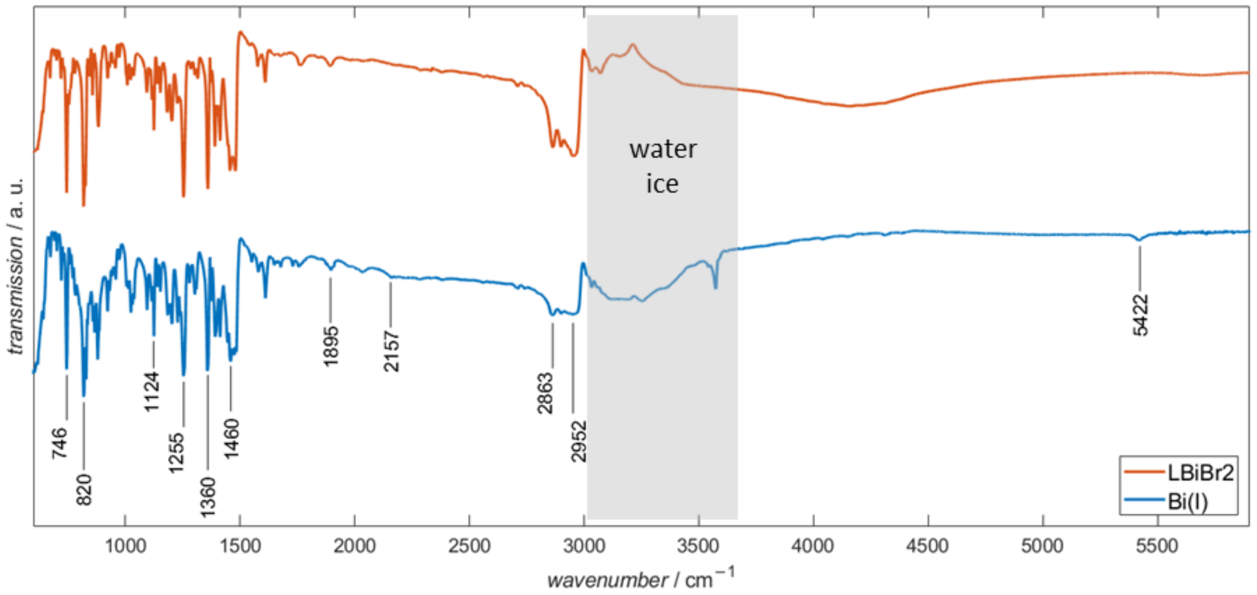

Figure S2. FTIR transmission spectra measured on powder samples of **1** (orange trace) and **2** (blue trace) at 10 K with a resolution of 2 cm^{-1}. During the measurements, water ice accumulated on the magnet windows lead to distortions in the spectral region from 3000 to 3700 cm^{-1} (gray shaded area), which is therefore discarded from further discussion. Spectra of **1** and **2** are vertically stacked for better visibility.

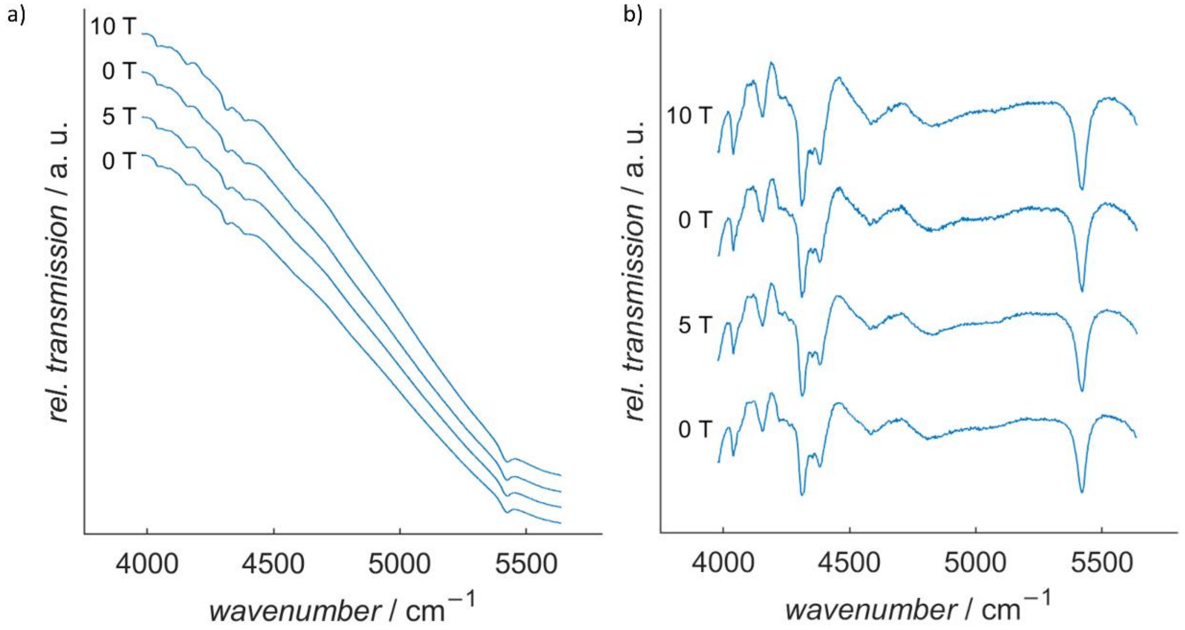

Figure S3. IR transmission spectra of **2** in the range between 3800 – 5700 cm^{-1} obtained at 10 K and the magnetic fields indicated in the figure, a) raw transmission IR spectra, b) transmission IR spectra after background correction by subtracting a polynomial function from the spectra depicted in the left panel.

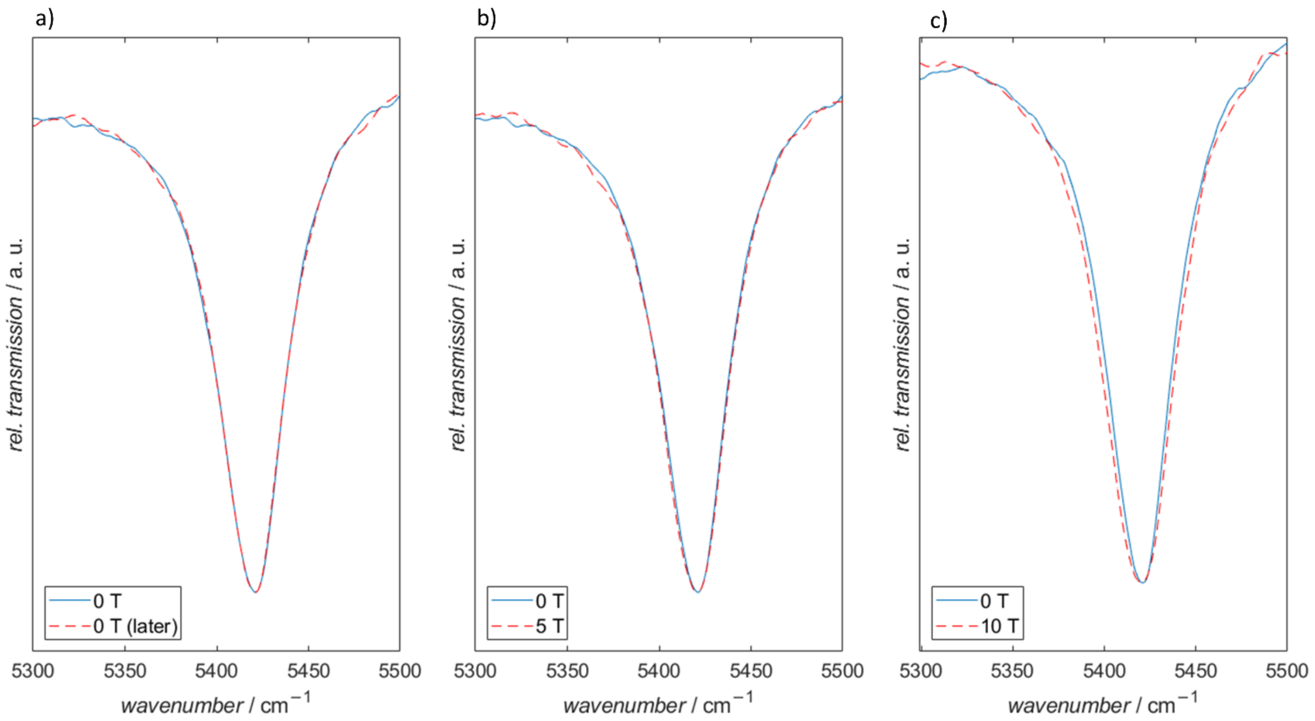

Figure S4. IR transmission spectra of **2** in the range between 5300 – 5500 cm^{-1}, a) Spectra obtained at 0 T with a time interval of about 5 hours (blue solid and red dashed lines, respectively), b) spectra taken at 0 T (blue solid line) and 5 T (red dashed line) c) spectra taken at 0 T (blue solid line) and 10 T (red dashed line). All spectra were obtained with an instrumental resolution of 2 cm^{-1} and at T = 10 K.

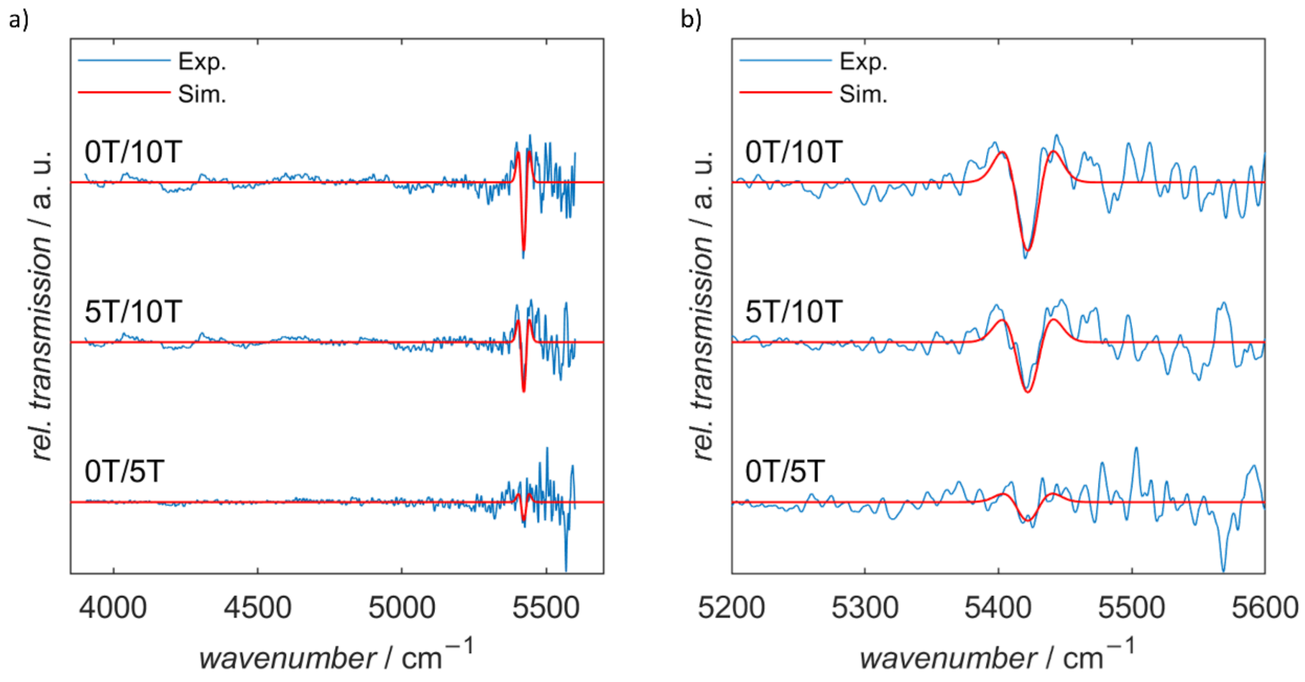

Figure S5. Experimental (blue) and simulated (red) 0 T/10 T, 5 T/10 T and 0 T/5 T IR-MDS of **2** taken at T = 10 K, plotted vs. energy ranges of a) 3800 – 5700 cm^{-1} and b) 5200 – 5600 cm^{-1}.

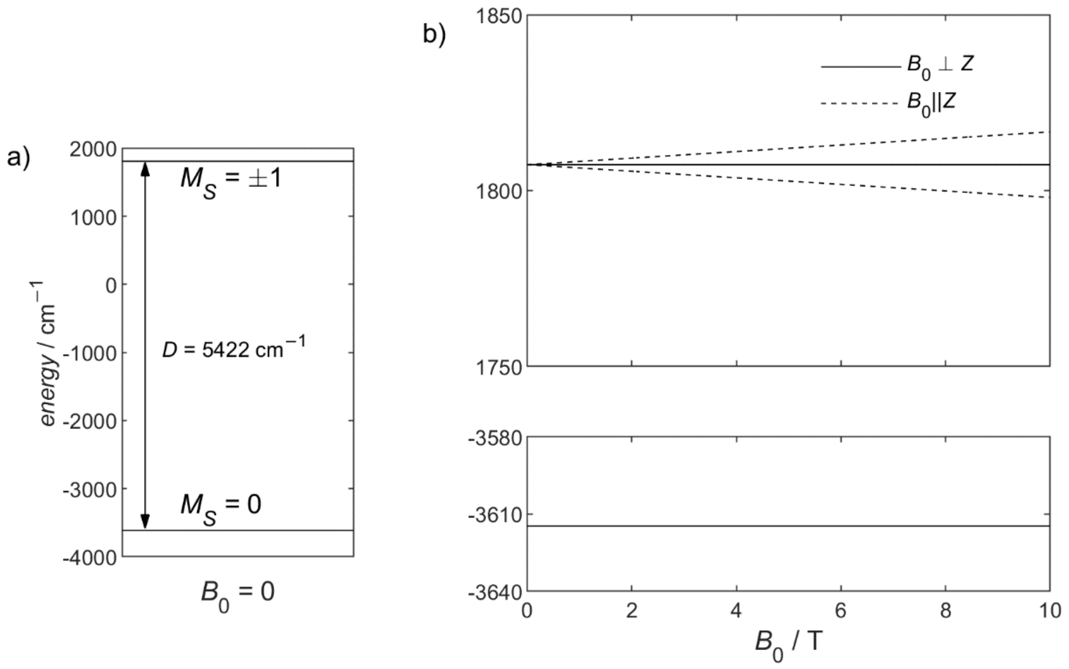

Figure S6. Calculated spin-energy levels with the spin Hamiltonian parameters for **2**. a) Zero-field magnetic sublevels: The splitting between the $M_S = 0$ and $M_S = \pm1$ energy levels corresponds to the axial ZFS parameter D. b) Magnetic field dependent spin energy levels for orientations of the ZFS tensor parallel and perpendicular to the external magnetic field B_0. The energy regions around the M_S levels are scaled for better visibility. Here, the $M_S = \pm1$ levels are split with increasing magnetic field by the Zeeman interaction. Depending on the orientation, the maximum splitting at 10 T is around 18 cm^{-1} and thus three orders of magnitude smaller than the splitting induced by the zero-field interaction, shown in subfigure a). The two allowed transitions between the $M_S = 0$ and $M_S = \pm1$ are thus energetically different at applied magnetic field. However, they are not resolved in the experiment due to D-strain and due to overlapping subspectra from the orientational disordered sample, and thus observed as field dependent line broadening.

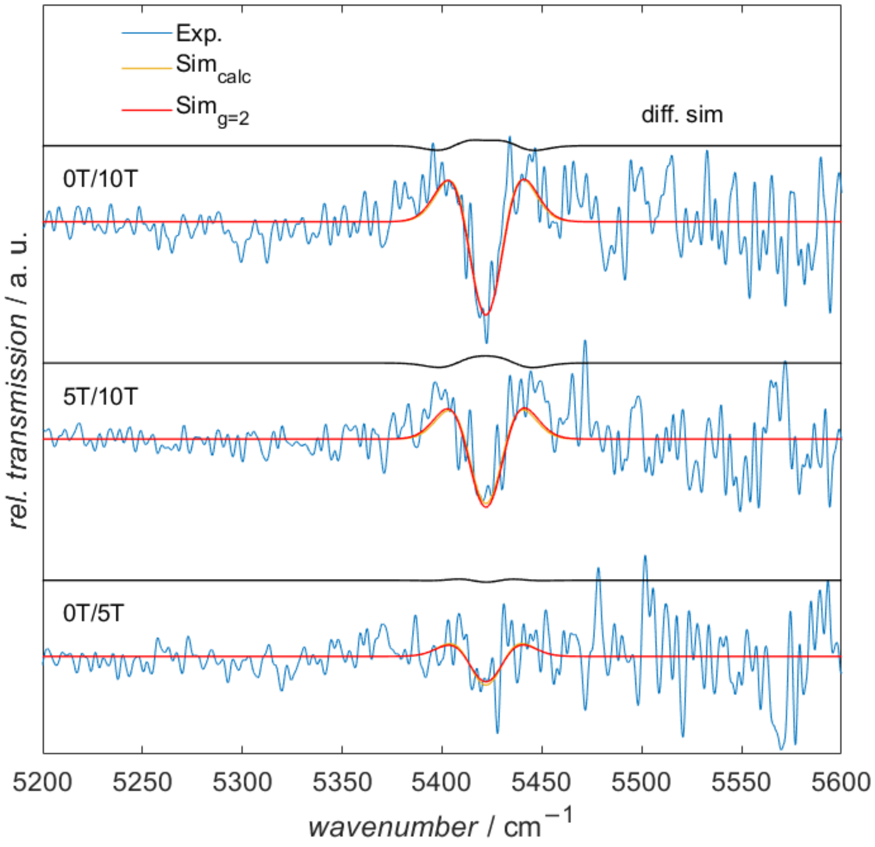

Figure S7. Experimental 0 T/10 T, 5 T/10 T and 0 T/5 T IR-MDS of **2** taken at $T = 10$ K (blue lines) vs. simulations obtained with $D = 5422$ cm^{-1}, $E = 0$ and the anisotropic matrix $\boldsymbol{g}$ = [1.77 1.84 1.98] obtained from the quantum chemical calculations (dark yellow lines) and an isotropic $g_{\mathrm{iso}} = 2$ (red lines). The differences between the two models are plotted in black.

4) Quantum Chemical Calculations

Wave function based *ab initio* calculations have been performed on **2** using the complete active space self-consistent field (CASSCF) followed by second-order N-Electron Valence perturbation theory (NEVPT2) calculations in conjunction with the X2C relativistic Hamiltonian as implemented in the ORCA package[8] (version 6.0). Spin-orbit coupling (SOC) was treated using the mean-field SOC Hamiltonian[9] including picture change effects. Further, details about the calculations are outlined in the supplemental material of Reference 1.

5) References

(1) Pang, Y.; Nöthling, N.; Leutzsch, M.; Kang, L.; Bill, E.; van Gastel, M.; Reijerse, E.; Goddard, R.; Wagner, L.; SantaLucia, D.; et al. Synthesis and isolation of a triplet bismuthinidene with a quenched magnetic response. *Science* **2023**, *380* (6649), 1043-1048. DOI: 10.1126/science.adg2833.
(2) Martin, W. C. Table of Spin-Orbit Energies for p-Electrons in Neutral Atomic (core)np Configurations. *J Res Natl Bur Stand A Phys Chem* **1971**, *75a* (2), 109-111. DOI: 10.6028/jres.075A.010.
(3) Akimov, A.; Masitov, A.; Korchagin, D.; Chapyshev, S.; Misochko, E.; Savitsky, A. W-band EPR studies of high-spin nitrenes with large spin-orbit contribution to zero-field splitting. *The Journal of Chemical Physics* **2015**, *143* (8). DOI: 10.1063/1.4929589.

(4) Akimov, A. V.; Ganushevich, Y. S.; Korchagin, D. V.; Miluykov, V. A.; Misochko, E. Y. The EPR Spectrum of Triplet Mesitylphosphinidene: Reassignment and New Assignment. *Angew. Chem., Int. Ed.* **2017**, *56* (27), 7944-7947. DOI: 10.1002/anie.201703629.
(5) Wu, M.; Li, H.; Chen, W.; Wang, D.; He, Y.; Xu, L.; Ye, S.; Tan, G. A triplet stibinidene. *Chem* **2023**, *9* (9), 2573-2584. DOI: 10.1016/j.chempr.2023.05.005.
(6) Wu, M.; Chen, W.; Wang, D.; Chen, Y.; Ye, S.; Tan, G. Triplet bismuthinidenes featuring unprecedented giant and positive zero field splittings. *Natl. Sci. Rev.* **2023**, *10* (10). DOI: 10.1093/nsr/nwad169.
(7) Nehrkorn, J.; Holldack, K.; Bittl, R.; Schnegg, A. Recent progress in synchrotron-based frequency-domain Fourier-transform THz-EPR. *J. Magn. Reson.* **2017**, *280*, 10-19. DOI: 10.1016/j.jmr.2017.04.001.
(8) Neese, F. Software update: The ORCA program system—Version 5.0. *WIREs Computational Molecular Science* **2022**, *12* (5), e1606. DOI: 10.1002/wcms.1606.
(9) Heß, B. A.; Marian, C. M.; Wahlgren, U.; Gropen, O. A mean-field spin-orbit method applicable to correlated wavefunctions. *Chem. Phys. Lett.* **1996**, *251* (5), 365-371. DOI: 10.1016/0009-2614(96)00119-4. Neese, F. Efficient and accurate approximations to the molecular spin-orbit coupling operator and their use in molecular g-tensor calculations. *The Journal of Chemical Physics* **2005**, *122* (3). DOI: 10.1063/1.1829047.
